# Microstructural abnormalities in the ATR and VOF underlie tone awareness deficits in Chinese children with developmental dyslexia: a DTI study

**DOI:** 10.3389/fpsyt.2026.1792594

**Published:** 2026-05-18

**Authors:** Simin Deng, Jiaxuan Fu, Xiaojing Song, Xinyun Lin, Xintong Su, Kaize Yang, Ranran Gong, Jingxian Zhao, Daosen Wang, Jie Tang, Si Tan, Xiuhong Li

**Affiliations:** 1 School of Public Health (Shenzhen), Shenzhen Campus of Sun Yat-sen University, Shenzhen, China; 2 Department of Rehabilitation Medicine, Dongguan Eighth People’s Hospital, Dongguan, China; 3 School of Public Health, Sun Yat-sen University, Guangzhou, China; 4 Qilu Hospital (Qingdao), Shandong University, Qingdao, China

**Keywords:** Chinese children, developmental dyslexia, diffusion tensor imaging (DTI), mediation effects, tone awareness

## Abstract

**Objective:**

Tone awareness is crucial for reading in Chinese children, significantly affecting those with developmental dyslexia (DD). This paper identifies microstructural abnormalities in the inferior fronto-occipital fasciculus (IFOF), uncinate fasciculus (UF), anterior thalamic radiation (ATR), and vertical occipital fasciculus (VOF) in Chinese children with DD and evaluates their potential relationships with tone awareness and DD.

**Methods:**

35 children with DD and 64 typically developing (TD) children were recruited from Guangdong Province, China. Diffusion tensor imaging (DTI) measured mean diffusivity (MD), axial diffusivity (AD), radial diffusivity (RD), and fractional anisotropy (FA). The Tone Awareness Judgment Task was used to measure tone awareness. Generalized linear regression and mediation models were used to explore associations and mediating effects.

**Results:**

Children with DD showed significantly lower MD, AD, and RD values in the bilateral IFOF and ATR, and right VOF compared to TD children, with no significant FA abnormalities. Limited associations between white matter microstructure and tone awareness were observed in the bilateral ATR and right VOF, but these associations did not survive multiple-comparison correction. Tone awareness mediated the relationships between microstructural abnormalities in the bilateral ATR and right VOF and DD.

**Conclusions:**

Microstructural abnormalities in the bilateral ATR and right VOF may be related to DD partly through tone awareness, although direct associations between tract microstructure and tone awareness were limited.

## 1 Introduction

Developmental dyslexia (DD) has been widely conceptualized within established theoretical frameworks, particularly the phonological deficit hypothesis, which posits that impairments in phonological processing constitute a core mechanism underlying reading difficulties across languages (1). In addition, pathway-based neurobiological models of language processing distinguish dorsal and ventral pathways supporting phonological decoding and lexical–semantic processing, respectively (2, 3). Tone awareness refers to an individual’s ability to perceive and control tonal features during tone production and perception (4). As a subcategory of phonological awareness in Chinese, it is a distinctive feature that distinguishes Chinese from alphabetic languages like English. For Chinese children, tone awareness is a crucial aspect of language processing, as tonal variations alter Chinese word meanings (4). Deficits in tone awareness significantly impair reading development, making its study in relation to developmental dyslexia (DD) essential.

DD is a specific learning disability characterized by severe reading difficulties despite normal intelligence and adequate educational opportunities (5). The prevalence of DD is approximately 5%-17% (6–8). Children with DD frequently encounter behavioral, academic, and psychological challenges, which persist into adulthood, leading to difficulties in employment and income issues, and affecting families and society (9, 10).

Research indicates that Chinese children with DD commonly struggle with tone awareness (11–13). The relationship between tone awareness deficits and DD in Mandarin versus alphabetic languages remains debated. Studies of phonological awareness in Mandarin Chinese have demonstrated that tone-related processing constitutes an important component of suprasegmental phonological skills and contributes to reading development in Chinese children (14, 15), although tone awareness tasks may also involve segmental phonological processing. Some studies argue that factors like grammar and vocabulary influence reading abilities (16), with Chinese reading disabilities may relate more to visual-spatial processing and memory than to tone awareness (17). Conversely, many studies emphasize the importance of tone awareness in Mandarin-speaking children, suggesting that tonal variations affect word meanings, leading to reading difficulties (18). Therefore, further studies are needed to explore the effect of tone awareness deficits on the Chinese DD and related brain mechanisms.

White matter transmits information between different brain regions, and its disruption can lead to DD. Investigating the white matter mechanisms underlying tone awareness deficits in Chinese children with DD is crucial, but it remains unclear which white matter integrity impacts tone awareness.

Diffusion Tensor Imaging (DTI) is a non-invasive imaging technique used to examine the microstructural properties of myelinated white matter tracts and brain connectivity. Common DTI parameters include Fractional Anisotropy (FA), Axial Diffusion (AD), Radial Diffusion (RD), and Mean Diffusion (MD) (19). FA represents the degree of anisotropy of water diffusion in biological tissues and is often interpreted as a quantitative biomarker of white matter “integrity” (20). AD indicates the rate of water diffusion along the main diffusion direction, while RD represents the rate of water diffusion perpendicular to the main diffusion direction. Changes in RD, without alterations in AD, are typically associated with myelin abnormalities, whereas changes in AD, without alterations in RD, are generally related to axonal diameter (21). MD reflects the overall degree of diffusion within the tissue and is defined as the mean of the three eigenvalues of the diffusion tensor (MD = (λ1 + λ2 + λ3)/3, or equivalently (AD + 2×RD)/3). Variations in MD may arise from multiple factors, including cell density, extracellular space, and other microstructural alterations.

Previous studies have identified several white matter tracts associated with language processing and reading abilities that may also contribute to tone awareness. In children with DD, structural impairments have been observed in the arcuate fasciculus and superior longitudinal fasciculus of the dorsal-sublexical pathway, as well as the inferior longitudinal fasciculus, inferior fronto-occipital fasciculus (IFOF), and uncinate fasciculus (UF) of the ventral-lexical pathway (22–26). While dorsal-stream pathways are primarily involved in segmental phonological decoding, tone awareness in the current study was hypothesized to place greater demands on auditory-visual integration and lexical-semantic processing. Accordingly, the present study focused on a broader set of ventral, occipito-temporal, and thalamo-cortical pathways, including the IFOF, UF, ATR, and VOF, rather than limiting the analysis to canonical language tracts alone (2, 3). Additionally, some studies have found abnormalities in the corpus callosum (27), thalamic radiation (28), and corona radiata (29). Compared to typically developing (TD) children, FA values in these tracts are consistently lower in children with DD, though the abnormalities in MD, AD, and RD remain inconclusive. Our previous research found a reduction in AD in the anterior thalamic radiation (ATR) of Chinese children with DD. Although numerous studies have examined phonological awareness and its neural correlates in dyslexia, including in tonal languages (30), the specific role of white matter pathways in tone awareness remains to be further clarified.

The IFOF and UF in the ventral-lexical pathway are crucial for semantic processing, particularly for understanding tone-related semantic information in Mandarin (24, 31–33). The synergistic action of the dorsal and ventral pathways facilitates rapid information transfer between brain regions involved in phonological and semantic processing, thereby promoting efficient reading (34). However, while dorsal-stream pathways are primarily implicated in segmental phonological decoding, tone awareness, which is closely related to suprasegmental phonological processing but may also involve segmental processing demands, is hypothesized to rely more strongly on ventral and occipito-temporal pathways involved in auditory-visual integration and lexical access. On this basis, we included not only ventral association tracts but also the ATR and VOF, as the present study aimed to capture a broader network potentially relevant to tone awareness rather than canonical language pathways alone. The vertical occipital fasciculus (VOF) is a major white matter tract connecting the dorsal and ventral language pathways (35), and functional magnetic resonance imaging (MRI) studies have shown that the VOF, connecting the parieto-occipital junction and occipito-temporal region, is related to tone awareness (2, 36, 37). Based on these findings, our study explores the relationship between these key white matter tracts and tone awareness in Chinese children with DD, selecting the IFOF, UF, ATR, and VOF as regions of interest (ROI).

We aim to determine whether these children exhibit structural abnormalities in these white matter tracts and whether these abnormalities may be related to tone awareness performance. In the present study, tone awareness is defined as the explicit discrimination of lexical tone categories across syllables, as measured by the tone awareness judgment task, rather than general tone perception or low-level auditory processing. By clarifying these relationships, our study seeks to provide new insights into the neural mechanisms underlying DD, which could inform the development of more effective diagnostic tools and targeted interventions, ultimately improving outcomes for children with DD.

## 2 Methods

### 2.1 Study participants

From July 2019 to October 2023, this study recruited right-handed children with normal (or corrected) vision and hearing from grades 2 to 5 in primary schools in Guangzhou and Shenzhen through online recruitment. Of the 118 participants who completed MRI scans, 19 were excluded due to missing images, excessive head movement, mixed-handedness, developmental delay, or missing test results. Leaving 99 children for analysis (35 DD children and 64 TD children).

DD children were diagnosed based on the Diagnostic and Statistical Manual of Mental Disorders, Fifth Edition (DSM-5) (5), using a comprehensive evaluation framework that integrated behavioral observation, standardized cognitive assessment, and detailed parental medical history, in accordance with established diagnostic practices for developmental dyslexia. Criteria included: 1) Literacy Test (38): Scoring 1.5 SD below the grade mean score; 2) Intelligence Test (39): Using the Chinese Revised Wechsler Intelligence Scale for Children, Fourth Edition (WISC-IV), with a full-scale intelligence quotient (FSIQ) ≥ 80 and a perceptual reasoning index ≥ 80; 3) Learning Disability Screening (40): Using the Learning Disability Screening Scale, with a total score < 65.

TD children met the following criteria: 1) Age-appropriate literacy skills, defined as Chinese character recognition performance within the normal range relative to grade-level norms and not meeting criteria for reading difficulty; 2) Intelligence Test (WISC-IV): FSIQ ≥ 80 and a perceptual reasoning index ≥ 80; 3) Learning Disability Screening: A total score ≥ 65.

Exclusion Criteria: 1) Neurodevelopmental disorders, including developmental delay, attention deficit hyperactivity disorder (ADHD), or tic disorders, as determined based on reported clinical diagnoses, developmental history obtained from structured parental interviews, and behavioral observation; 2) Medical implants or claustrophobia contraindicating MRI; 3) Left-handed or mixed-handed children; 4) Failure to pass MRI quality checks or diffusion weighted imaging (DWI) head motion parameters exceeding 2 SD above the mean (41).

This study was approved by the Ethics Committee of the School of Public Health, Sun Yat-sen University (Approval No. 2016-036). All participants and their guardians signed informed consent forms.

### 2.2 Tone awareness test

The Tone Awareness Judgment Task, developed by Dandan Shen from Nanjing Normal University, used materials from the Chinese textbooks for grades 1 to 3 by Guangdong Educational Publishing House Edition (42). The task involved high-frequency, daily-use characters. The examiner played an audio of three Chinese characters, where two characters had the same tone, and one had a different tone. The participant identified the character with a different tone. For example, “1) shui3, 2) zui3, 3) gui1, gui’s tone is different, select 3).” The task included 15 formal items and 2 practice items. Each formal item scored 1 point, for a total of 15 points. Higher scores indicated better tone awareness.

### 2.3 Diagnostic assessment tests

#### 2.3.1 Literacy test

The standardized literacy test developed by Wang Xiaoling et al. assessed the reading level of Chinese primary school students (38). It was completed within 50 minutes under parental supervision, without discussion or reference materials. Scores were based on the number of correct characters and grade-level difficulty coefficients. The criterion-related validity coefficient is 0.98, and the test-retest reliability is 0.98 (38). This test was used both to identify children with reading difficulty in the DD group and to confirm age-appropriate literacy performance in the TD group based on grade-level norms.

#### 2.3.2 Intelligence test

The WISC-IV assessed the intelligence of children aged 6 to 16 (39), including 10 core and 4 supplemental subtests, providing a FSIQ and four indices (Working Memory Index, Processing Speed Index, Verbal Comprehension Index, and Perceptual Reasoning Index). Administered by experienced researchers in a quiet environment, excluding children with a FSIQ below 80.

#### 2.3.3 Learning disability screening

The Pupil Rating Scale Revised Screening for Learning Disabilities (PRS) was completed by homeroom teachers with at least 3 months of student contact (40). It consists of 24 items, with lower scores indicating more severe learning disabilities. Screening criteria include a verbal score below 20, a non-verbal score below 40, and a total score below 65. The test-retest reliability is 0.92, and the Cronbach’s alpha coefficient is 0.95 (43).

### 2.4 MRI scanning

#### 2.4.1 Data collection

The Siemens Skyra 3.0T MRI scanner from Guangzhou Panorama Medical Imaging Diagnostic Center was used. Parents played pre-recorded MRI videos and noise audio at home to help children adapt. During the scan, children remained relaxed and still, with foam pads and earplugs to reduce discomfort, and falling asleep was allowed.

#### 2.4.2 Scanning parameters

1) T1-weighted Images: TE = 2.19 ms, TR = 1800 ms, flip angle = 9°, matrix = 256×256, slices = 176, slice thickness = 1.0 mm, voxel size = 1.0×1.0×1.0 mm³, FOV = 256×256×176 mm, scan time ~7.42 minutes. 2) DTI Images: TR = 10000 ms, TE = 92 ms, slices = 75, slice thickness = 2 mm, b-value = 1000 s/mm², voxel size = 2.0×2.0×2.0 mm³, matrix = 256×256, FOV = 256×256×75 mm, 64 directions, scan time ~11.22 minutes.

### 2.5 Preprocessing

DICOM images were converted to nii format using the dcm2niigui tool (https://www.nitrc.org/projects/mricron/) and inspected for quality by three analysts. FSL software (44) version 6.0 (FMRIB Software Library, https://fsl.fmrib.ox.ac.uk/fsl/) was used for DTI image processing, including: 1) Removal of non-brain tissues and creation of whole-brain masks; 2) Image cropping; 3) Head motion and eddy current correction; 4) Data averaging; 5) Calculation of diffusion tensor parameters. Head motion was calculated using the method described by Power et al (41), with participants excluded if motion exceeded 2 SD above the mean.

### 2.6 DTI diffusion parameters calculation

**Figure 1:**
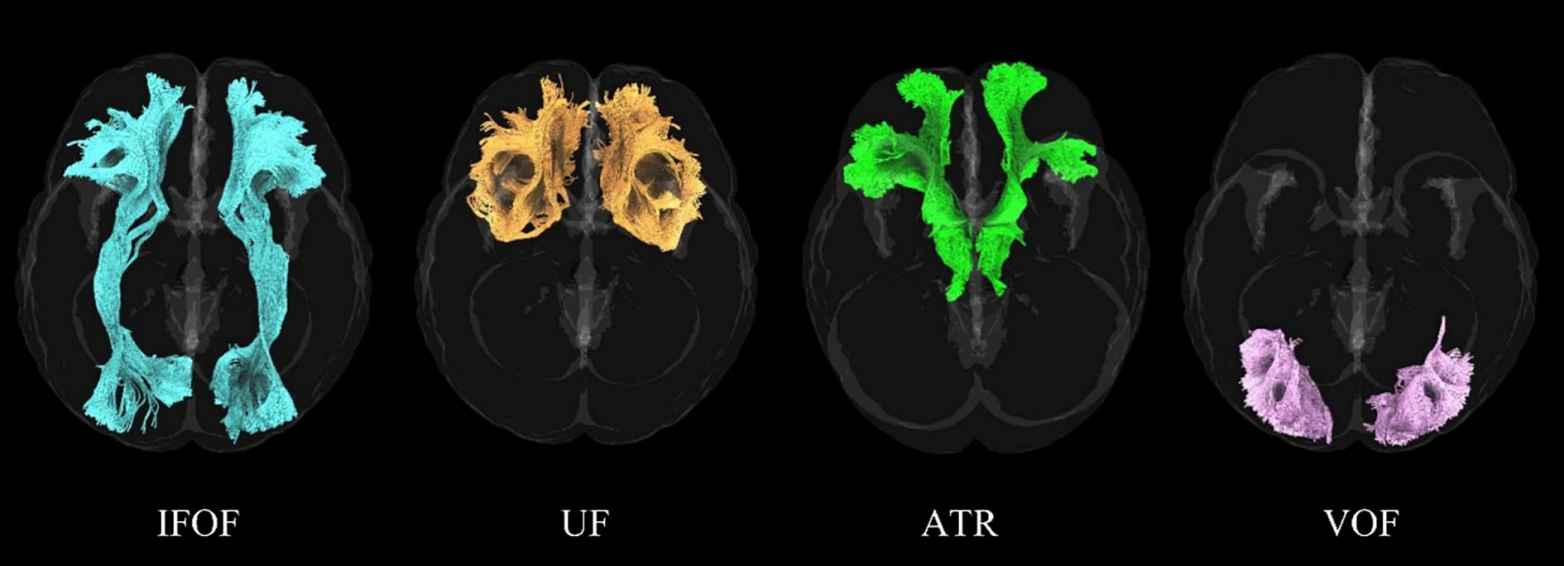
Fiber bundles of interest. This figure illustrates the fiber bundles under study. IFOF, inferior fronto-occipital fasciculus; UF, uncinate fasciculus; ATR, anterior thalamic radiation; VOF, vertical occipital fasciculus.

Automated fiber tracking was performed using DSI Studio (https://dsi-studio.labsolver.org/). Images were non-linearly registered to the MNI152NLin2009cAsym template (45) to ensure all data were in the same standard space. The accuracy of the b-table direction was checked by comparing it to the population average template. Deterministic fiber tracking algorithms were used, with enhanced tracking strategies to improve reproducibility. Anisotropy thresholds were defined within empirically constrained ranges recommended by the DSI Studio software and previous methodological studies, angular thresholds were set between 15° and 90°, and step sizes were set between 0.5 and 1.5 voxels. Fibers with lengths <44.6385 mm or >252.851 mm were excluded. These lower and upper fiber-length thresholds were automatically derived from the tract-length distribution estimated by the DSI Studio software and were used to exclude anatomically implausible streamlines. The same thresholds were applied consistently across all participants, and the reported numerical precision reflects software-generated values rather than biologically meaningful cutoffs. Using these settings, the interested fiber bundles (bilateral IFOF, VOF, UF, and ATR, **Figure 1**) were reconstructed for each participant. MD, AD, RD, and FA values were extracted for subsequent statistical analysis.

### 2.7 Statistical analysis

Data were entered using Epidata 3.1 and analyzed using R 4.1.1. Approximately 1% of missing values (demographic and behavioral variables only) were imputed using the “Mice” package to avoid subject exclusion; sensitivity analyses confirmed consistent results when these cases were omitted. Independent t-tests compared age and tone awareness scores between groups, while chi-square tests compared gender, parental education level, and family monthly income per capita. All statistical analyses were two-tailed, with α = 0.05. Independent t-tests were used as an initial exploratory step to assess MD, AD, RD, and FA values of bilateral IFOF, VOF, UF, and ATR between DD and TD groups. Fiber bundle indices showing significant group differences were subsequently entered into regression analyses for further examination while controlling for potential confounders.

Generalized Linear Models (GLMs) examined the association between fiber bundles and DD, and between fiber bundles and tone awareness, with fiber bundle indices as independent variables and group status or tone awareness scores as dependent variables. Age, gender, and whole-brain head motion were included as covariates to control for potential confounding effects. Mean whole-brain head motion did not differ significantly between the DD and TD groups. Sensitivity analyses were conducted using a stricter head motion exclusion criterion, and the main results remained consistent, indicating that the findings were not driven by motion-related artifacts. The fiber bundle indices related to both DD and tone awareness were included in further mediation analyses.

In the mediation analysis, fiber bundle indices were specified as independent variables, tone awareness scores as the mediator, and group status (DD vs. TD) as the dependent variable. Given the binary nature of the outcome variable, mediation analyses were conducted within a generalized modeling framework rather than assuming a standard linear outcome model. Mediation and outcome models were constructed using the”Mediation”package, with all models adjusted for age, gender, and whole-brain head motion. Bootstrapping with 5000 resamples was used to ensure robust estimation of indirect effects. Excess relative risk (ERR) was used to assess the total effect (TE) of fiber bundles on DD, which was decomposed into direct effects and mediation effects (46). ERR was chosen because it facilitates additive decomposition of effects in the context of a binary outcome, allowing clear partitioning of total, direct, and mediation effects. The mediation proportion indicated the proportion of the total effect explained by mediation. All continuous variables were standardized in regression and mediation analyses.

To control the Type I error rate arising from multiple comparisons, false discovery rate (FDR) correction was applied using the Benjamini-Hochberg procedure. FDR correction was performed separately within each diffusion metric (FA, MD, AD, and RD) across all examined tracts and hemispheres, rather than across all tests simultaneously. This strategy was chosen because FA, MD, AD, and RD reflect related yet partially distinct aspects of white matter microstructure and were therefore treated as separate families of hypotheses, while also balancing sensitivity and control of false discoveries.

Following prior neuroimaging studies, multiple nominal q-value thresholds (q = 0.05, 0.10, and 0.25) were examined to reflect different levels of tolerance for false discoveries. A more stringent threshold (e.g., q = 0.05) yields fewer significant findings, whereas more liberal thresholds (e.g., q = 0.25) allow detection of potentially meaningful effects. For transparency, both uncorrected p-values and FDR-adjusted q-values are reported in the Results and corresponding tables. In addition, interpretation of the findings emphasizes effect sizes and 95% confidence intervals (CIs), rather than relying solely on statistical significance.

## 3 Results

### 3.1 Demographic characteristics of the two groups of children

The average age of children in the DD group was 9.76 ± 1.07 years, with 77.14% of the DD children being boys. Compared to the TD group, the DD group had significantly lower literacy levels. However, there were no statistically significant differences between the two groups in terms of age, gender, parental education level, and average monthly household income. (**Table 1**).

**Table 1:** Demographic characteristics of the DD and TD children.

Characteristics	Means (SD)/N (%)			*P* value
	Total (N = 99)	DD (N = 35)	TD (N = 64)	
**Age**	9.56 (1.09)	9.76 (1.07)	9.45 (1.09)	0.18
Sex				0.21
Boy	67 (67.67%)	27 (77.14%)	40 (62.50%)	
Girl	32 (32.32%)	8 (22.86%)	24 (37.50%)	
Maternal education level				0.09
Below Bachelor	38 (39.58%)	17 (53.13%)	21 (32.81%)	
Bachelor’s degree or above	58 (60.42%)	15 (46.88%)	43 (67.19%)	
Paternal education level				0.06
Below Bachelor	36 (37.50%)	17 (53.13%)	19 (29.69%)	
Bachelor’s degree or above	60 (62.50%)	15 (46.88%)	45 (70.31%)	
Family economic situation				0.71
≤ ￥5000/month	16 (18.82%)	4 (14.81%)	12 (20.69%)	
￥5000-15000/month	42 (49.41%)	15 (55.56%)	27 (46.55%)	
> ￥15000/month	27 (31.76%)	8 (29.63%)	19 (32.76%)	
Mean FD	0.34 (0.19)	0.36 (0.20)	0.33 (0.18)	0.50
Tone awareness	12.58 (3.24)	10.62 (3.97)	13.65 (2.13)	**<0.001**

DD, developmental dyslexia; TD, typically developing; SD, standard deviation; FSIQ, full-scale intelligence quotient; FD, framewise displacement; significant *p* values were in bold.

### 3.2 Comparison of tone awareness between groups

**Figure 2:**
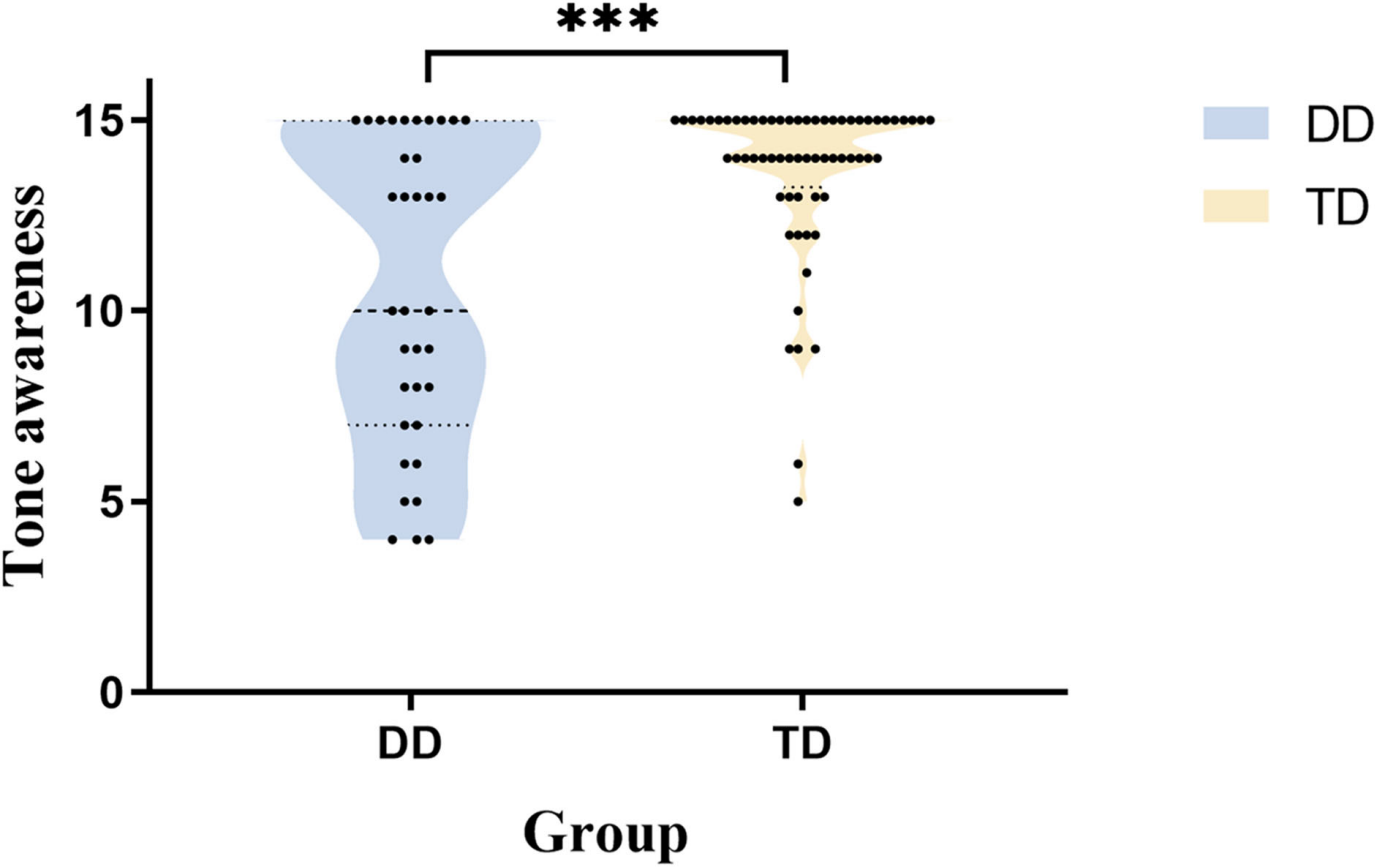
Comparison of tone awareness between children with DD and TD. ***p < 0.001. DD, developmental dyslexia; TD, typically developing.

The results indicate that, after controlling for demographic factors such as age and gender, the tone awareness scores of children in the DD group were significantly lower than those in the TD group, suggesting a tone awareness deficit in DD children. (**Figure 2**).

### 3.3 Comparison of DTI metrics of white matter tracts between groups

**Figure 3:**
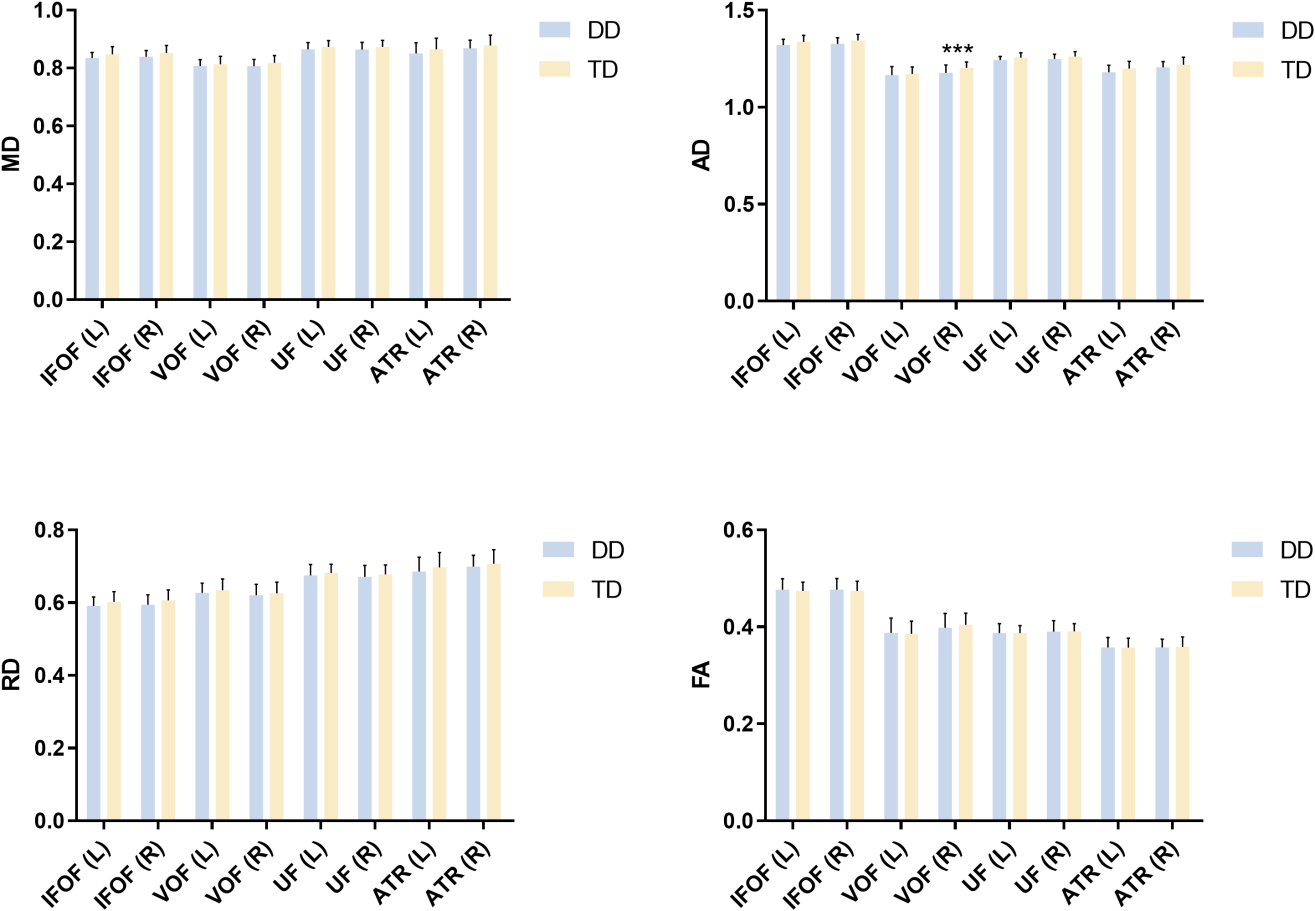
Comparison of DTI metrics of white matter tracts between children with DD and TD. The MD values of the bilateral IFOF and the right VOF, the AD values of the bilateral IFOF, UF, ATR, and the right VOF, as well as the RD values of the bilateral IFOF, were significantly lower in the DD group compared to the TD group. ***p < 0.001. DD, developmental dyslexia; TD, typically developing; IFOF_L, left inferior fronto-occipital fasciculus; IFOF_R, right inferior fronto-occipital fasciculus; VOF_L, left vertical occipital fasciculus; VOF_R, right vertical occipital fasciculus; UF_L, left uncinate fasciculus; UF_R, right uncinate fasciculus; ATR_L, left anterior thalamic radiation; ATR_R, right anterior thalamic radiation.

This study selected the bilateral IFOF, VOF, UF, and ATR as the tracts of interest. Independent sample t-tests were conducted on the DTI metrics of these tracts between the two groups, with age, gender, and head motion parameter mean framewise displacement (FD) as covariates. The findings indicate that the MD values of the bilateral IFOF and the right VOF, the AD values of the bilateral IFOF, UF, the right VOF, and the left ATR, as well as the RD values of the bilateral IFOF, were lower in the DD group than in the TD group. Among these findings, the MD values of the bilateral IFOF and the AD value of the right VOF remained significant after FDR correction, whereas the remaining group differences were significant only at the uncorrected level. (**Table 2**, **Figure 3**).

**Table 2:** The comparison of DTI metrics of each ROI between children with DD and TD children.

Tracts	DD (*M ± SD*)	TD (*M ± SD*)	Total (*M ± SD*)	*P value*
MD				
IFOF (L)	0.834 (0.019)	0.846 (0.026)	0.842 (0.024)	0.014*
IFOF (R)	0.838 (0.021)	0.852 (0.025)	0.847 (0.024)	0.007**
VOF (L)	0.806 (0.022)	0.813 (0.027)	0.811 (0.025)	0.233
VOF (R)	0.806 (0.023)	0.818 (0.024)	0.813 (0.024)	0.019*
UF (L)	0.870 (0.022)	0.873 (0.021)	0.864 (0.023)	0.070
UF (R)	0.869 (0.023)	0.872 (0.022)	0.863 (0.024)	0.084
ATR (L)	0.850 (0.035)	0.864 (0.038)	0.859 (0.037)	0.093
ATR (R)	0.867 (0.028)	0.877 (0.036)	0.874 (0.033)	0.166
AD				
IFOF (L)	1.321 (0.029)	1.336 (0.034)	1.331 (0.033)	0.025*
IFOF (R)	1.327 (0.030)	1.344 (0.031)	1.338 (0.031)	0.011*
VOF (L)	1.166 (0.043)	1.171 (0.036)	1.169 (0.038)	0.492
VOF (R)	1.177 (0.040)	1.202 (0.030)	1.193 (0.036)	<0.001***+++
UF (L)	1.251 (0.023)	1.255 (0.024)	1.243 (0.018)	0.008**
UF (R)	1.257 (0.025)	1.261 (0.025)	1.249 (0.023)	0.019*
ATR (L)	1.180 (0.035)	1.199 (0.037)	1.192 (0.038)	0.019*
ATR (R)	1.205 (0.030)	1.219 (0.037)	1.214 (0.035)	0.054
RD				
IFOF (L)	0.590 (0.025)	0.602 (0.028)	0.598 (0.027)	0.050*
IFOF (R)	0.594 (0.027)	0.606 (0.028)	0.602 (0.028)	0.043*
VOF (L)	0.627 (0.026)	0.634 (0.030)	0.631 (0.029)	0.270
VOF (R)	0.620 (0.029)	0.625 (0.030)	0.623 (0.030)	0.396
UF (L)	0.679 (0.026)	0.681 (0.023)	0.675 (0.029)	0.242
UF (R)	0.675 (0.027)	0.677 (0.026)	0.671 (0.030)	0.249
ATR (L)	0.685 (0.039)	0.696 (0.041)	0.692 (0.040)	0.207
ATR (R)	0.699 (0.030)	0.706 (0.038)	0.704 (0.036)	0.325
FA				
IFOF (L)	0.476 (0.022)	0.474 (0.018)	0.474 (0.019)	0.531
IFOF (R)	0.476 (0.023)	0.474 (0.019)	0.475 (0.020)	0.635
VOF (L)	0.387 (0.030)	0.385 (0.025)	0.386 (0.027)	0.703
VOF (R)	0.398 (0.029)	0.404 (0.023)	0.402 (0.026)	0.270
UF (L)	0.414 (0.022)	0.409 (0.023)	0.411 (0.023)	0.282
UF (R)	0.386 (0.019)	0.389 (0.018)	0.388 (0.019)	0.528
ATR (L)	0.387 (0.016)	0.387 (0.015)	0.387 (0.019)	0.977
ATR (R)	0.390 (0.018)	0.390 (0.016)	0.390 (0.022)	0.986

*p<0.05 (uncorrected); **p<0.01 (uncorrected); ***p<0.001 (uncorrected). FDR correction was performed separately within each diffusion metric. Results surviving FDR correction at q<0.05 are indicated by +++. DD, developmental dyslexia; TD, typical developmental; MD, mean diffusivity; AD, axial diffusivity; RD, radial diffusivity; FA, fractional anisotropy; IFOF, inferior frontal occipital fasciculus; VOF, vertical occipital fasciculus; UF, uncinate fasciculus; ATR, anterior thalamic radiation; L, left; R, right. Significant p values were in bold

### 3.4 Association of abnormal white matter tracts with DD and tone awareness

Based on independent sample t-test results, the MD values of the bilateral IFOF and right VOF, the AD values of the bilateral IFOF, UF, ATR, and right VOF, and the RD values of the bilateral IFOF were selected as independent variables. A generalized linear regression model was constructed with group and tone awareness scores as dependent variables, and age, gender, and mean FD as covariates. The results indicate that these DTI metrics are negatively correlated with DD after adjusting for covariates. Additionally, the MD and AD values of the right VOF and the AD values of the bilateral ATR are positively correlated with tone awareness, with no significant group differences. (**Table 3**).

**Table 3:** Effects of DTI metrics of each ROI associated with tone awareness and Chinese developmental dyslexia risk.

Tracts	Tone awareness		Chinese developmental dyslexia risk	
	β (95%CI)	*P value*	OR (95%CI)	*P value*
MD				
IFOF (L)	0.592 (-0.080, 1.264)	0.087	0.879 (0.805, 0.955)	0.004**+++
IFOF (R)	0.612 (-0.048, 1.272)	0.072	0.875 (0.799, 0.947)	0.003**+++
VOF (L)	0.713 (0.054, 1.372)	0.036*	0.948 (0.864, 1.038)	0.230
VOF (R)	0.836 (0.168, 1.504)	0.016*	0.893 (0.810, 0.982)	0.021*
UF (L)	0.187 (-0.495, 0.869)	0.113	0.917 (0.836, 1.014)	0.090
UF (R)	0.272 (-0.425, 0.969)	0.446	0.921 (0.840, 1.027)	0.126
ATR (L)	0.497 (-0.166, 1.161)	0.145	0.914 (0.802, 1.007)	0.074
ATR (R)	0.694 (0.032, 1.355)	0.042*	0.927 (0.848, 1.027)	0.139
AD				
IFOF (L)	0.511 (-0.133, 1.154)	0.123	0.898 (0.820, 0.987)	0.025*
IFOF (R)	0.546 (-0.097, 1.190)	0.099	0.882 (0.803, 0.969)	0.013*
VOF (L)	0.277 (-0.388, 0.942)	0.416	0.955 (0.875, 1.067)	0.356
VOF (R)	0.718 (0.058, 1.378)	0.035*	0.866 (0.783, 0.945)	0.002**+++
UF (L)	0.301 (-0.354, 0.955)	0.370	0.890 (0.811, 0.964)	0.009**
UF (R)	0.391 (-0.261, 1.044)	0.242	0.899 (0.820, 0.982)	0.019*
ATR (L)	0.702 (0.063, 1.341)	0.033*	0.876 (0.786, 0.985)	0.022*
ATR (R)	0.771 (0.129, 1.414)	0.020*	0.895 (0.817, 0.990)	0.031*
RD				
IFOF (L)	0.479 (-0.205, 1.164)	0.173	0.903 (0.819, 0.987)	0.028*
IFOF (R)	0.499 (-0.186, 1.183)	0.156	0.903 (0.820, 0.981)	0.020*
VOF (L)	0.759 (0.096, 1.423)	0.027*	0.964 (0.874, 1.062)	0.403
VOF (R)	0.585 (-0.097, 1.267)	0.095	0.960 (0.874, 1.070)	0.437
UF (L)	0.098 (-0.591, 0.786)	0.781	0.946 (0.858, 1.048)	0.269
UF (R)	0.151 (-0.554, 0.856)	0.675	0.955 (0.867, 1.062)	0.360
ATR (L)	0.360 (-0.317, 1.036)	0.299	0.934 (0.824, 1.033)	0.212
ATR (R)	0.576 (-0.094, 1.247)	0.095	0.951 (0.868, 1.054)	0.301
FA				
IFOF (L)	-0.136 (-0.814, 0.543)	0.696	1.039 (0.940, 1.176)	0.467
IFOF (R)	-0.221 (-0.919, 0.477)	0.537	1.030 (0.934, 1.166)	0.553
VOF (L)	-0.564 (-1.239, 0.110)	0.104	0.996 (0.909, 1.135)	0.974
VOF (R)	-0.159 (-0.845, 0.526)	0.649	0.946 (0.852, 1.061)	0.324
UF (L)	-0.027 (-0.705, 1.585)	0.652	0.995 (0.901, 1.115)	0.980
UF (R)	0.032 (-0.655, 1.501)	0.719	0.983 (0.892, 1.104)	0.758
ATR (L)	0.094 (-0.615, 0.802)	0.796	0.994 (0.899, 1.119)	0.948
ATR (R)	-0.066 (-0.746, 0.614)	0.849	0.974 (0.890, 1.081)	0.632

*p<0.05 (uncorrected); **p<0.01 (uncorrected). FDR correction was performed separately within each diffusion metric. Results surviving FDR correction at q<0.05 are indicated by +++. MD, mean diffusivity; AD, axial diffusivity; RD, radial diffusivity; FA, fractional anisotropy; IFOF, inferior frontal occipital fasciculus; VOF, vertical occipital fasciculus; UF, uncinate fasciculus; ATR, anterior thalamic radiation; L, left; R, right. Significant p values were in bold.

### 3.5 Mediating role of tone awareness in the association between white matter tracts and DD

**Figure 4:**
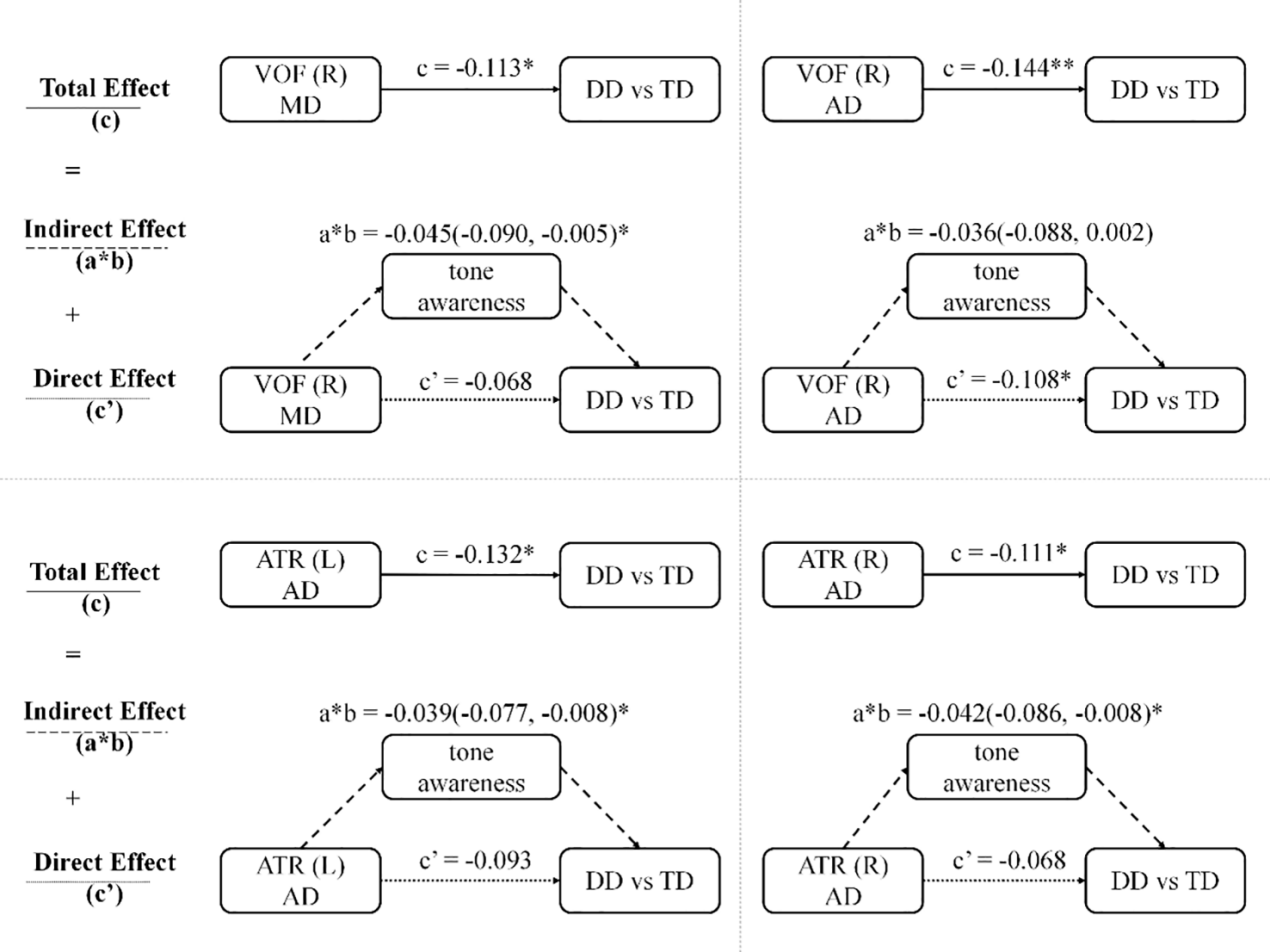
The mediating role of tone awareness in the relationship between white matter tracts and developmental dyslexia. Tone awareness significantly mediated the relationship between the MD value of the right VOF and DD, as well as the AD values of the bilateral ATR and DD. The mediation effect values were -0.045 (95% CI: -0.090, -0.005) for the right VOF, -0.039 (95% CI: -0.077, -0.008) for the left ATR, and -0.111 (95% CI: -0.202, -0.010) for the right ATR, accounting for 39.6%, 29.8%, and 38.4% of the total effect, respectively. DD, developmental dyslexia; TD, typically developing; VOF, vertical occipital fasciculus; ATR, anterior thalamic radiation; R, right; L, left. *p<0.05 (uncorrected); **p<0.01 (uncorrected).

Based on the generalized linear regression model, DTI metrics related to both tone awareness and DD (including the MD and AD values of the right VOF and the AD values of the bilateral ATR) were selected as independent variables, tone awareness scores as the mediator, with age, gender, and mean FD as covariates, and group as the dependent variable to construct mediation and outcome models. The results indicate that these DTI metrics significantly affect DD with a negative correlation. Notably, tone awareness significantly mediated the relationship between the MD value of the right VOF and DD, and the AD values of the bilateral ATR and DD, with mediation effect values of -0.045 (95% CI: -0.090, -0.005), -0.039 (95% CI: -0.077, -0.008), and -0.111 (95% CI: -0.202, -0.010), accounting for 39.6%, 29.8%, and 38.4% of the total effect, respectively. (**Table 4**, **Figure 4**).

**Table 4:** The mediation effects of tone awareness on the associations of DTI metrics with Chinese developmental dyslexia risk.

Tracts	Total effect	Mediation effect	Direct effect	Proportion of mediation
	ERR (95% CI)	ERR (95% CI)	ERR (95% CI)	
VOF (R) MD	-0.113(-0.211, -0.018)*+++	-0.045(-0.090, -0.005)*+++	-0.068(-0.157, 0.014)	39.6%*
VOF (R) AD	-0.144(-0.245, -0.057)**+++	-0.036(-0.088, 0.002)	-0.108(-0.198, -0.025)*	25.1%
ATR (L) AD	-0.132(-0.241, -0.015)*+++	-0.039(-0.077, -0.008)*+++	-0.093(-0.200, 0.020)	29.8%*
ATR (R) AD	-0.111(-0.202, -0.010)*+++	-0.042(-0.086, -0.008)*+++	-0.068(-0.156, 0.027)	38.4%*

*p<0.05 (uncorrected); **p<0.01 (uncorrected). FDR correction was performed separately within each diffusion metric. Results surviving FDR correction at q<0.05 are indicated by +++. MD, mean diffusivity; AD, axial diffusivity; VOF, vertical occipital fasciculus; ATR, anterior thalamic radiation; L, left; R, right. Significant p values were in bold.

## 4 Discussion

This study is the first to explore the relationship between disrupted white matter microstructural alterations in the IFOF, UF, ATR, and VOF and tone awareness in Chinese children with DD. We found that the DD group had significantly lower tone awareness and significantly reduced MD and AD values in the right VOF, which remained significant after FDR correction. Associations between tone awareness and the right VOF and bilateral ATR were observed only at the uncorrected level, whereas no significant association was found for the bilateral IFOF. None of these tract-tone associations survived FDR correction. Notably, among the examined tracts, alterations in the right VOF showed the most consistent and statistically robust effects, particularly in relation to DD, as they survived FDR correction across group comparisons and mediation models. In contrast, direct associations involving other tracts, such as the ATR, were observed primarily at the uncorrected level and should therefore be interpreted as exploratory. Moreover, mediation analyses indicated that tone awareness significantly mediated the relationship between DD and the right VOF (MD/AD) and bilateral ATR (AD). Our findings provide new insights into the connection between specific white matter tract abnormalities and tone awareness deficits in Chinese children with DD, with the most robust evidence observed in the right VOF and bilateral IFOF, while findings involving other tracts should be regarded as exploratory.

### 4.1 The abnormal white matter tracts in Chinese DD children

This study found structural abnormalities in white matter tracts in children with DD, consistent with previous research. However, unlike studies reporting reduced FA in specific tracts such as the left IFOF (47), the bilateral VOF and the left UF (48), and the bilateral ATR (28), we observed no significant differences between DD and TD children. This suggests that group differences in DD may be reflected in DTI metrics such as MD, AD, and RD rather than FA values. However, alterations in these diffusion metrics do not map directly onto specific pathological mechanisms and may arise from multiple microstructural factors, including axonal density, extracellular space, and fiber geometry.

Specifically, we observed significant reductions in MD and AD in the right VOF, and these findings survived FDR correction, identifying the VOF as a robust marker of abnormal white matter in Chinese DD children. Reductions observed in other tracts, including the bilateral IFOF, UF, and ATR, did not survive FDR correction and were therefore considered secondary and exploratory findings rather than primary evidence of white matter abnormalities. Other reductions in the bilateral IFOF, UF, and ATR were observed at the uncorrected level but did not survive FDR correction and should therefore be interpreted with caution. While FA is a common marker of white matter integrity (19), changes in RD (without AD changes) are linked to myelin abnormalities, whereas changes in AD (without RD changes) are linked to axon diameter alterations (21). MD reflects the overall degree of diffusion freedom in the tissue. Lower FA is usually driven by macroscopic factors (e.g., crossing fibers) (49), implying that our findings may reflect microstructural alterations rather than macroscopic ones, as FA is influenced by both microscopic (myelination, axon characteristics) and macroscopic (axon coherence) factors (50). Due to study population heterogeneity, sample size, and statistical analysis methods, further research is needed to validate these findings.

The IFOF is involved in semantic memory and control, with the right IFOF supporting automated processing and the left IFOF supporting controlled processing (51). Direct electrical stimulation of the right IFOF may cause semantic association disorders (52). Myelin and axon abnormalities in the bilateral IFOF of DD may affect both modes of semantic processing. The UF supports semantic information retrieval (53) and, with the IFOF, forms the ventral language pathway, essential for sound-to-meaning mapping and tone understanding. The VOF connects the visual word form area with the dorsal parietal-occipital region (54, 55), and its abnormalities may disrupt visual processing, hindering the early reading information transformation. Disruptions in the VOF may also impair tonal information processing. Abnormalities in the ATR, which connects the thalamus to the frontal cortex and is involved in declarative memory and motor control (56, 57), may cause difficulties in mastering the form, sound, and meaning of Chinese characters in DD children, as the role of ATR is crucial for accurate pronunciation and associating tones with meanings.

### 4.2 Association between abnormal white matter tracts and tone awareness in Chinese DD children

This study used a generalized linear regression model to explore the association between abnormal white matter tracts and tone awareness in Chinese DD children. Results showed limited positive associations between tone awareness and the MD and AD values of the right VOF and the AD values of the bilateral ATR at the uncorrected level, whereas no significant association was found for the bilateral IFOF. None of these tract-tone associations survived FDR correction. In contrast, the bilateral ATR also showed positive correlations with tone awareness, but these did not survive FDR correction and should be interpreted with caution. The VOF connects the dorsal and ventral language pathways, supporting rapid information transfer for phonological and semantic processing, crucial for reading (34, 35). Tone awareness is crucial in distinguishing characters and determining meaning in Chinese, suggesting that the VOF’s role is in tone awareness processing (58). The robust associations observed in the bilateral IFOF and right VOF suggest that these tracts may serve as stable neural correlates of tone awareness deficits in Chinese DD. In contrast, associations involving the ATR did not survive correction and should be regarded as preliminary, warranting further investigation in larger samples.

The ATR links the thalamus to the frontal cortex and the anterior cingulate cortex (57) and is involved in executive functions related to declarative memory (59). Although the ATR also showed positive associations with tone awareness, these findings did not survive FDR correction and therefore should be regarded as preliminary.

### 4.3 Mediating role of tone awareness in the association between white matter tracts and DD

This study explored the potential mediating mechanisms between white matter tracts and DD in children, using DTI indices of fiber tracts related to tone awareness and DD as independent variables, tone awareness scores as the mediator, and group (DD and TD) as the dependent variable. Results showed that tone awareness significantly mediated the relationship between DD and the MD and AD values of the right VOF, as well as the AD values of the left and right ATR, and these mediation effects survived FDR correction. Given the limitations of DTI scalar metrics, which can be influenced by crossing fibers, partial volume effects, and noise (49), the observed mediation effects should not be interpreted as evidence of specific pathological processes. Rather, they indicate that microstructural variations within these tracts are statistically associated with tone awareness and DD status. Accordingly, tone awareness may represent a possible behavioral process that partially accounts for the association between microstructural alterations in the VOF and ATR and DD, although alternative explanations cannot be excluded within the present cross-sectional design. In addition, because the tone awareness task used in this study may involve both segmental and suprasegmental phonological processing, the findings should be interpreted as reflecting tone awareness performance rather than a pure suprasegmental construct. Finally, the tract selection in the present study was hypothesis-driven but not exhaustive. Although we focused on the IFOF, UF, ATR, and VOF to capture pathways potentially relevant to tone awareness, other tracts, such as the ILF, were not examined and should be considered in future studies.

This study supports clinical interventions to improve tone awareness and alleviate DD symptoms, but further research is needed to validate these findings and explore potential biological mechanisms. The case-control design of this study cannot determine the temporal relationship, and future research should adopt prospective designs to establish causality. Additionally, the heterogeneity in tone awareness among Chinese DD children was not classified due to the limited sample size and lack of classification thresholds. Therefore, it remains unclear whether the observed effects apply uniformly across the DD group or are driven primarily by a subgroup of children with more pronounced tone awareness impairment. Future studies should classify the types of tone awareness deficits in a larger sample and examine within-group variability more directly to further verify the relationship between tone awareness levels, white matter tracts, and network topological properties in Chinese children with DD.

## 5 Conclusion

This study provides new insights into the neural mechanisms of tone awareness, offering empirical evidence that structural abnormalities in white matter tracts may underlie tone awareness deficits in Chinese children with DD. Significant tract abnormalities associated with DD were observed in several white matter tracts, with FDR-corrected effects identified in the bilateral IFOF for MD and in the right VOF for AD. Tone awareness showed limited associations with the right VOF and bilateral ATR at the uncorrected level, whereas no significant association was found for the bilateral IFOF. None of these tract-tone associations survived FDR correction. Tone awareness further mediated the relationship between DD and the right VOF (MD/AD) and bilateral ATR (AD), with these effects surviving FDR correction. By linking these abnormalities to DD through tone awareness, the study advances our understanding of the cognitive processes involved in language processing and reading development. However, the present findings more strongly support associations between these tracts and DD itself than with tone awareness per se, and therefore do not justify interpreting the IFOF and VOF as stable neural markers of tone awareness deficits.

## Data Availability

The raw data supporting the conclusions of this article will be made available by the authors, without undue reservation.
